# Predicting Adverse Childhood Experiences from Family Environment Factors: A Machine Learning Approach

**DOI:** 10.3390/bs15091216

**Published:** 2025-09-08

**Authors:** Nii Adjetey Tawiah, Emmanuel A. Appiah, Felisha White

**Affiliations:** 1 College of Humanities, Education and Social Sciences, Delaware State University, 1200 N. DuPont Highway, Dover, DE 19901, USA; 2 Department of Mathematics, Prairie View A&M University, Prairie View, TX 77446, USA; emappiah@pvamu.edu; 3 Institutional Effectiveness, Spelman College, 350 Spelman Lane S.W., Atlanta, GA 30314, USA; felishawhite@spelman.edu

**Keywords:** adverse childhood experiences, family environment, machine learning, predictive modeling, data analysis

## Abstract

Adverse childhood experiences (ACEs) are associated with profound long-term health and developmental consequences. However, current identification strategies are largely reactive, often missing opportunities for early intervention. Therefore, the potential of machine learning to proactively identify children at risk of ACE exposure needs to be explored. Using nationally representative data from 63,239 children in the 2018–2020 National Survey of Children’s Health (NSCH) after listwise deletion, we trained and validated multiple machine learning models to predict ACE exposure categorized as none, one, or two or more ACEs. Model performance was assessed using accuracy, precision, recall, F1 scores, and area under the curve (AUC) metrics with 5-fold cross-validation. The Random Forest model achieved the highest predictive accuracy (82%) and demonstrated strong performance across ACE categories. Key predictive features included child sex (female), food insufficiency, school absenteeism, quality of parent–child communication, and experiences of bullying. The model yielded high performance in identifying children with no ACEs (F1 = 0.89) and moderate performance for those with multiple ACEs (F1 = 0.64). However, performance for the single ACE category was notably lower (F1 = 0.55), indicating challenges in predicting this intermediate group. These findings suggest that family environment factors can be leveraged to predict ACE exposure with clinically meaningful accuracy, offering a foundation for proactive screening protocols. However, implementation must carefully address systematic selection bias, clinical utility limitations, and ethical considerations regarding predictive modeling of vulnerable children.

## 1. Introduction

The National Survey of Children’s Health (NSCH), funded and directed by the Health Resources and Services Administration (HRSA) Maternal and Child Health Bureau (MCHB), provides critical data for studying the association between Adverse Childhood Experiences (ACEs) and various health outcomes in children in the United States (Child and Adolescent Health Measurement Initiative [CAHMI], 2021). This contemporary research builds upon the foundational work of Felitti, Anda and colleagues, who initially published the ACE framework in 1998 from a landmark study examining the relationship between adverse childhood experiences and adult health risk factors and disease conditions (Felitti et al., 1998). This research has had a profound impact on public health theory and policy at both national and global levels (Anda et al., 2010; Hillis et al., 2004), demonstrating “a consistently strong relationship between an increasing number of ACEs and poor health outcomes in adults” (Felitti et al., 1998).

### 1.1. Family Functioning and Environment: The Influence on Adverse Childhood Experiences

In the context of ACEs, family functioning serves as a crucial lens for examining both trauma transmission mechanisms and pathways for cultivating resilience in children and adolescents. Research indicates strong associations between ACEs, family functioning, and mental health problems, with demographic factors mediating these relationships (Scully et al., 2019; Walsh, 2016).

The quality of family interactions, communication patterns, and emotional support can either amplify or mitigate the potential impact of traumatic experiences (Murphey & Sacks, 2019). High-quality family functioning characterized by effective parent–child communication and reduced parental stress serves as a protective mechanism for adolescent development, associated with improved mental health, enhanced school engagement, and reduced risks of substance misuse (Balistreri & Alvira-Hammond, 2016).

### 1.2. Research Gap and Objective

While ACEs have been extensively studied as traumatic events with lasting health impacts, significant gaps remain in understanding how family environments influence ACE transmission and effects. Current research provides insufficient examination of family context and dynamics that either perpetuate or disrupt intergenerational trauma cycles (Balistreri & Alvira-Hammond, 2016; Mistry et al., 2002; Fan & Chen, 2012).

Traditional statistical approaches often struggle to capture the non-linear and multidimensional interactions present in family systems data. Machine learning methods offer advantages for pattern recognition, handling complex relationships, and developing predictive models that can identify at-risk families before adverse childhood experiences occur or accumulate (Chen & Guestrin, 2016; Hastie et al., 2009).

This study examines whether specific family environment characteristics can predict ACE exposure, offering several advantages: early identification enabling preventive interventions; targeted resource allocation to highest-risk families; pattern recognition of subtle relationships; and development of personalized intervention approaches based on family risk profiles.

## 2. Methodology

### 2.1. Data Source and Study Population

The National Survey of Children’s Health (NSCH) is a comprehensive, nationally representative survey conducted annually by the U.S. Census Bureau in collaboration with the Health Resources and Services Administration’s Maternal and Child Health Bureau (Child and Adolescent Health Measurement Initiative [CAHMI], 2021). This analysis utilized combined data from the 2018–2020 NSCH surveys, providing a robust sample of children aged 0–17 years across all 50 states and the District of Columbia.

### 2.2. Sample Selection and Missing Data Analysis

The combined 2018–2020 NSCH dataset initially included 132,173 children. After excluding cases with missing ACE data, 130,037 children remained. A comprehensive missing data analysis revealed systematic patterns related to child age and survey design, with school-related variables (bullying, academic engagement, school safety) only administered to children aged 6–17 years, resulting in systematic “missing” data for younger children (28% of the sample).

We employed listwise deletion, retaining only cases with complete data across all variables used in the analysis, resulting in a final analytical sample of 63,239 children (47.8% of the original sample). While this approach reduces sample size, it ensures model training on complete information and avoids imputation assumptions that may not hold across diverse family contexts.

The analytical sample showed systematic differences from the full sample, with significantly lower ACE prevalence (31% vs. 38% with any ACEs), indicating systematic bias toward families with more stable survey participation patterns. This selection bias toward more stable family environments potentially limits generalizability to the most vulnerable populations experiencing the highest levels of adversity.

### 2.3. Outcome and Predictor Variables

The primary outcome (ACE2more) was constructed as a three-level categorical measure: (1) experiencing no ACEs, (2) experiencing 1 ACE, and (3) experiencing 2 or more ACEs.

Predictors were systematically selected from four theoretically grounded domains based on established ACE literature and social determinants of health framework. Demographic variables included child age, sex, race, and family composition characteristics that have been consistently associated with ACE risk in previous research. Socioeconomic factors encompassed measures of family cohesion, parental health status, and child behavioral indicators that reflect household stability and stress levels. Community and school environment variables captured neighborhood safety perceptions, school engagement metrics, and peer relationship quality as indicators of broader ecological influences on child development. Family functioning measures included communication patterns between parents and children, shared family activities, and other protective behaviors that may buffer against adverse experiences or promote resilience.

### 2.4. Data Preprocessing, and Feature Optimization

Our data preprocessing followed a systematic protocol to ensure data quality and model performance. Missing data handling procedures addressed the systematic missing value patterns present in the NSCH dataset, where values 90, 95, 96, and 99 representing missing responses were transformed to NaN and subsequently dropped to maintain data integrity and prevent bias in model training. This approach ensured that machine learning models operated on reliable inputs without the confounding effects of incomplete data.

Variable scaling procedures were implemented to address the heterogeneous nature of the dataset, with continuous variables standardized using z-score transformation to prevent any variable from dominating the model due to scale differences. This standardization was essential given the diverse measurement scales across demographic, behavioral, and environmental variables.

Categorical encoding protocols converted all categorical variables using one-hot encoding to enable machine learning algorithm processing while preserving the categorical nature of the data without imposing artificial ordinal relationships. This approach was particularly important given the numerous categorical variables characterizing family structure, communication patterns, and community engagement.

Feature selection procedures systematically organized variables into theoretically meaningful categories including emotional and mental health indicators, community and school activities, family and health activities, and child and family demographics. Selection was guided by established social determinants of health theory and domain expertise, emphasizing the crucial role of theoretical knowledge in extracting meaningful patterns from complex family systems data.

### 2.5. Machine Learning Model Development

We systematically evaluated ten machine learning algorithms to capture diverse patterns in family environment–ACE relationships. Given that family systems involve complex, non-linear interactions that traditional statistical methods may not fully capture, we selected algorithms representing different analytical approaches: traditional statistical methods for baseline comparison, tree-based ensemble methods for capturing non-linear interactions, and instance-based learning for local pattern recognition.

Among traditional models, logistic regression was selected for its interpretability and ability to estimate odds of ACE exposure based on family environment factors (Tabachnick & Fidell, 2019). This provides a familiar statistical foundation that allows behavioral scientists to interpret odds ratios while establishing baseline performance for comparison with more complex algorithms. Linear discriminant analysis provided a method to distinguish multiple family environments and identify pattern recognition capabilities for modeling relationships between ACEs and risk stratification utilizing linear combinations that best separate the groups (McLachlan, 2004; Hastie et al., 2009). Support vector machine was employed to create optimal boundaries between ACE categories, particularly effective for complex or non-linear patterns and separating different family environment categories with maximum-margin boundaries (Cortes & Vapnik, 1995; Schölkopf & Smola, 2002).

Tree-based models formed the core of our ensemble approach. Decision tree algorithms were chosen for their interpretability and visualization capabilities, effectively displaying how different ACE factors lead to specific outcomes (Breiman et al., 1984; Quinlan, 1986). Random Forest was implemented for classification while controlling overfitting, increasing accuracy, and ranking the most important ACE predictors through bootstrap aggregation (Breiman, 2001; Liaw & Wiener, 2002). This approach is particularly valuable for family environment data because it can identify complex interactions between multiple risk factors while providing interpretable feature importance rankings. Gradient boosting methods, including XGBoost, enabled learning of complex patterns and relationships in the data, facilitating correction of prediction errors and proving effective for capturing cumulative and compounding effects of multiple ACEs on lifelong health trajectories (Chen & Guestrin, 2016). Light Gradient Boosting Machine was implemented to predict ACE-related outcomes while handling missing data, incorporating techniques such as leaf-wise tree growth and exclusive feature bundling to enhance model accuracy and efficiency (Ke et al., 2017). CatBoost was utilized specifically to handle the numerous categorical variables in the dataset, processing categorical variables natively without requiring extensive preprocessing, thereby reducing risk of data leakage and enhancing model accuracy (Prokhorenkova et al., 2018). These sequential learning approaches are especially suited to ACE prediction because they can capture the cumulative and compounding nature of family stressors over time.

Finally, K-Nearest Neighbors was incorporated as an instance-based learning approach, predicting a child’s family environment status by comparing their ACE profile to those of similar cases with known outcomes, providing a non-parametric alternative to the other modeling approaches (Cover & Hart, 1967; Hughes et al., 2017).

This multi-algorithm approach enables identification of the most appropriate analytical framework for family environment–ACE relationships while providing cross-validation of findings across different modeling assumptions, essential for establishing robust predictions in complex family systems.

### 2.6. Model Training and Validation

Stratified 5-fold cross-validation ensured balanced representation of ACE categories across training and validation sets. Hyperparameters were optimized using grid search with cross-validation. The natural class distribution was preserved during training, with evaluation emphasizing class-specific performance metrics.

Model performance was assessed using multiple metrics: accuracy, precision, recall, F1-scores, and area under ROC curve. Random Forest was selected as the optimal model based on overall classification accuracy, balanced performance across ACE categories, clinical interpretability, and robustness across cross-validation folds.

## 3. Results

### 3.1. Sample Characteristics

The analytical sample comprised 63,239 children after listwise deletion. The sample was evenly distributed by sex (52.0% male, 48.0% female) and included children across all age groups: 28.0% aged 0–5 years, 30.7% aged 6–11 years, and 41.3% aged 12–17 years.

### 3.2. ACE Distribution

Table 1 presents the analytical sample; 69.0% of children had experienced no ACEs, 18.8% had experienced one ACE, and 12.3% had experienced two or more ACEs. This distribution indicates that approximately one in three children had experienced at least one adverse childhood experience.

### 3.3. Comparison with Full Sample

The analytical sample showed systematically lower ACE prevalence compared to the full sample with available ACE data (31% vs. 38% with any ACEs), indicating potential selection bias toward more stable family environments.

### 3.4. Family Environment Characteristics

We employed bivariate analysis to reveal systematic relationships between family environment variables and ACE exposure. While overall ACE prevalence showed minimal differences between males (31% with any ACEs) and females (31.5% with any ACEs), child sex (female) emerged as the strongest predictor in the machine learning models. This apparent contradiction suggests that while overall exposure rates are similar, there may be differential patterns in the types of ACEs experienced, reporting behaviors, or co-occurring risk factors between genders that enhance predictive importance beyond simple prevalence rates.

Most families (74.0%) reported ability to afford adequate food, while 22.5% experienced some level of food insufficiency. Food insufficiency showed strong associations with higher ACE exposure (Gundersen & Ziliak, 2015; Chilton et al., 2017). Clear trends emerged between school absenteeism and ACE exposure (Blodgett & Lanigan, 2018). The association between adverse childhood experience (ACE) and school success in elementary. As missed school days increased, the proportion of children with no ACE exposure steadily declined (Stempel et al., 2017). Interestingly, children not enrolled in school had a high rate (65.8%) of no exposure to ACEs, although this may reflect other factors such as homeschooling or age.

**Figure 1:**
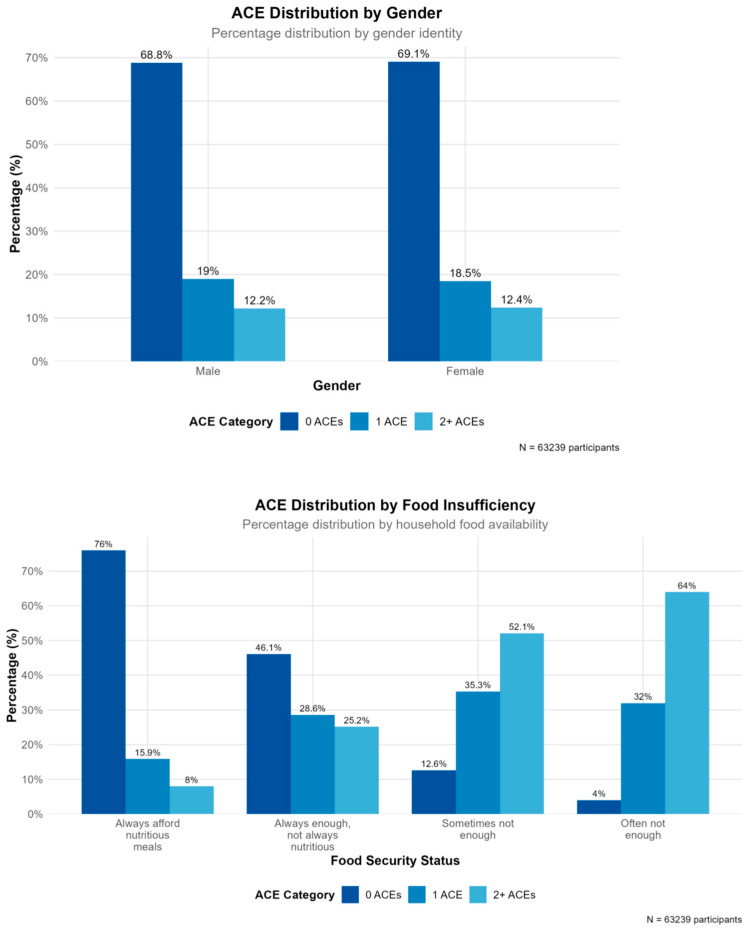

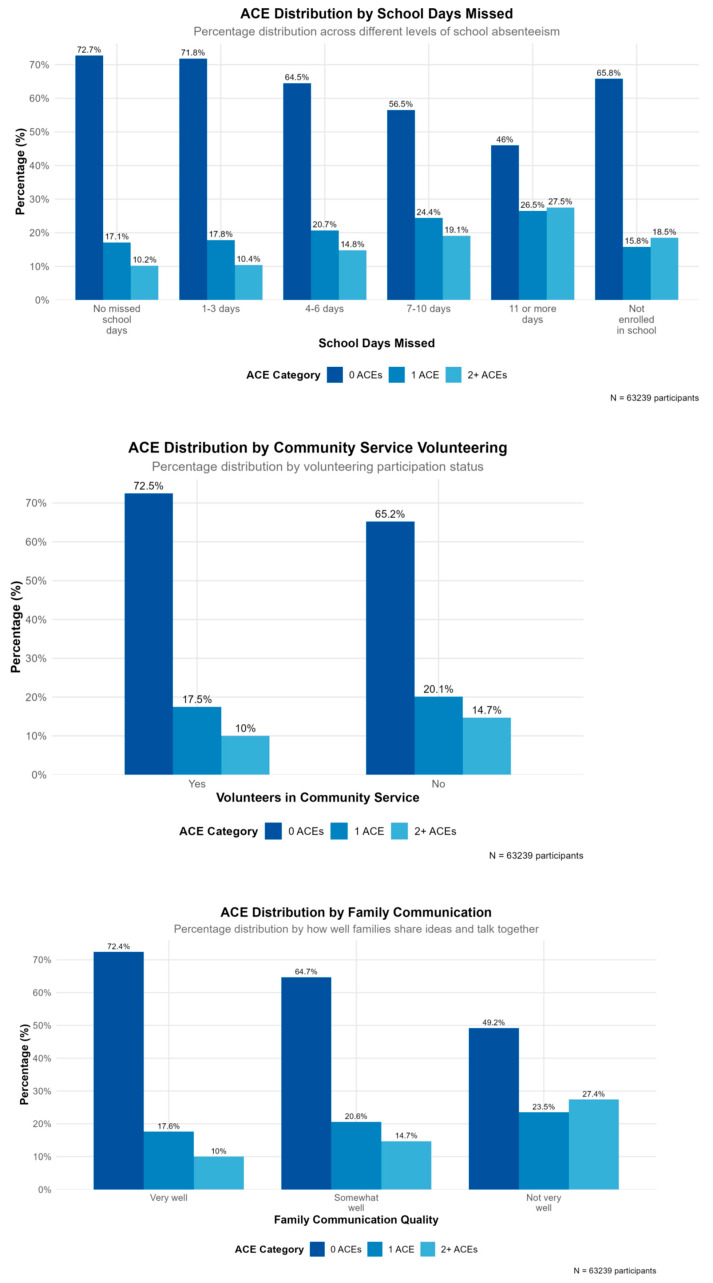

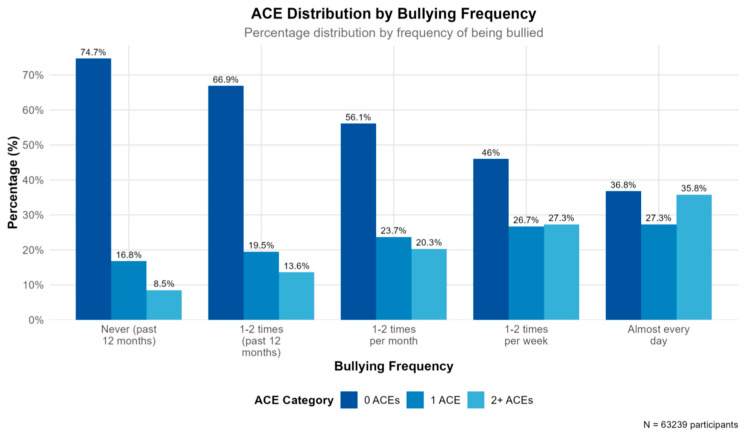
The most influential family environment variables.

Families reporting regular shared meals and open parent–child communication demonstrated lower rates of ACE exposure. Children engaged in volunteer or community service activities were less likely to report higher levels of adverse childhood experiences (Balistreri & Alvira-Hammond, 2016; Bethell et al., 2014). Parent–child communication quality emerged as a significant protective factor. Among school-age children, most demonstrated positive academic engagement, though chronic absenteeism showed strong associations with higher ACE exposure. A complete overview of the bivariate analysis is presented in Figure 1.

### 3.5. Machine Learning Model Performance

Random Forest achieved the highest predictive accuracy (82%) among all tested algorithms, with a macro-averaged F1-score of 0.69 and micro-averaged F1-score of 0.82.

Class-specific performance revealed important patterns across the three ACE categories. Children with no ACEs demonstrated excellent model performance with precision of 0.81, recall of 0.98, F1-score of 0.89, and AUC of 0.89, indicating the model’s strong ability to correctly identify low-risk children. The single ACE category presented significant challenges for prediction, achieving precision of 0.88 but recall of only 0.40, resulting in an F1-score of 0.55 and AUC of 0.84. Children with two or more ACEs showed moderate performance with precision of 0.81, recall of 0.53, F1-score of 0.64, and AUC of 0.91.

The notably poor performance for the single ACE category (F1 = 0.55) indicates significant challenges in predicting this intermediate group, which has important implications for clinical screening applications where accurate identification of all risk levels is essential.

**Figure 2:**
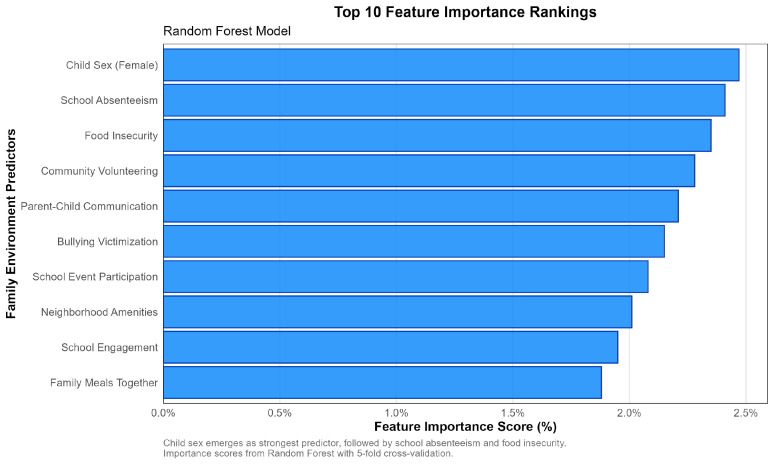
Random Forest Classifier Results: Top 10 Feature Importance Rankings.

Random Forest feature importance analysis revealed a clear hierarchy of predictive factors for ACE exposure (Figure 2). Child sex (female) emerged as the strongest predictor, followed by school absenteeism as a major behavioral indicator. Food insufficiency represented a critical socioeconomic risk factor, while community volunteering participation served as an important protective factor. Parent–child communication quality functioned as a key family functioning indicator, and bullying victimization reflected significant peer relationship challenges. School event participation indicated community engagement levels, while neighborhood amenities represented environmental factors. School engagement served as an academic risk factor, and family meals together provided an indicator of family cohesion and stability.

### 3.6. Multiclass ROCAUC Analysis

**Figure 3:**
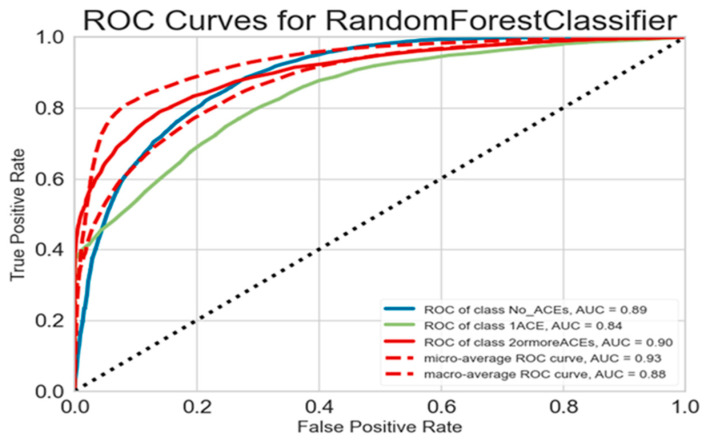
ROC Curves for Random Forest Classifier.

Figure 3 presents strong predictive performance of the Random Forest Classifier in distinguishing Adverse Childhood Experience (ACE) categories, with the highest class-specific ROC-AUC observed for individuals with two or more ACEs (AUC = 0.91), followed by those with no ACEs (AUC = 0.89) and one ACE (AUC = 0.84) (Figure 3). While the model showed excellent overall accuracy, the slightly lower performance for the one ACE group suggests potential overlap in feature patterns. Aggregate metrics further confirmed the model’s robustness, with a micro-average ROC-AUC of 0.93 and a macro-average of 0.88, indicating both high overall accuracy and balanced classification across categories. These results underscore the model’s utility in ACE prediction, particularly for identifying individuals with higher exposure, and highlight opportunities for refinement through feature enhancement or advanced learning techniques.

### 3.7. Precision-Recall Curve Analysis for Random Forest Classifier

**Figure 4:**
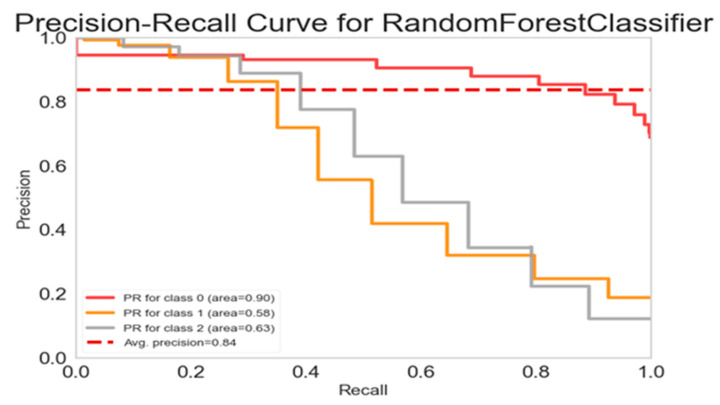
Precision-Recall Curve for Random Forest Classifier.

The Random Forest classifier exhibited differential precision and recall across ACE categories (Figure 4). Precision was highest for individuals with no ACEs (0.90), but notably lower for those with one ACE (0.57) and two or more ACEs (0.63), suggesting challenges in accurately distinguishing individuals with any ACE exposure. These disparities may be attributed to overlapping feature distributions or class imbalance. Despite this, the model achieved an average precision (AP) score of 0.84, indicating strong overall classification performance. While the classifier demonstrates reliable accuracy in identifying individuals without ACEs, enhancing precision for the one or multiple ACE groups may require rebalancing techniques, feature optimization, or hyperparameter adjustments. The model’s robust AP score supports its potential for early risk detection and targeted intervention.

### 3.8. Confusion Matrix Analysis

**Figure 5:**
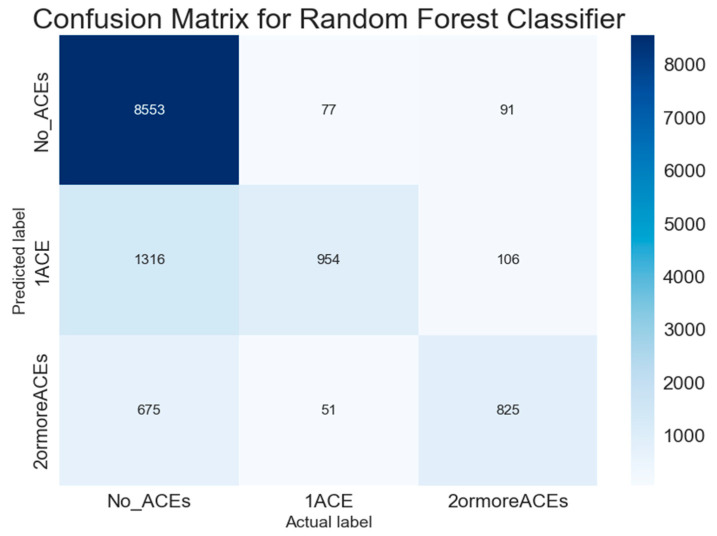
Confusion Matrix for Random Forest Classifier showing classification accuracy across ACE categories. Values represent actual counts of cases, with diagonal values indicating correct classifications. The matrix reveals excellent performance for identifying children with no ACEs (98.1% sensitivity) but substantial challenges in accurately classifying children with any ACE exposure.

The confusion matrix for the Random Forest classifier reveals important classification patterns across ACE categories (Figure 5). The model demonstrates exceptional specificity for identifying children with no ACEs, correctly classifying 8553 of 8721 cases (98.1% sensitivity), with only 168 cases misclassified into ACE exposure categories.

However, performance varies dramatically across risk categories. For children with single ACE exposure, the model correctly identified only 954 of 2376 cases (40.2% sensitivity), with 1316 cases (55.4%) misclassified as having no ACEs and 106 cases (4.5%) misclassified as having multiple ACEs. This pattern suggests substantial overlap in family environment characteristics between the single ACE group and both other categories.

For children with multiple ACEs, the model achieved moderate sensitivity (825 of 1551 cases, 53.2% correctly classified), with 675 cases (43.5%) misclassified as having no ACEs and 51 cases (3.3%) misclassified as single ACE exposure. The high rate of false negatives (children with ACEs classified as having none) is particularly concerning for clinical applications where missing at-risk children represents the most serious error type.

These patterns confirm the heterogeneous nature of the single ACE category and highlight the model’s primary utility in confirming low-risk status rather than comprehensively identifying all children who might benefit from intervention.

### 3.9. Model Comparison

To evaluate model performance in predicting adverse childhood experiences (ACEs) within the family environment, a comparative analysis was conducted using multiple multiclass and advanced tree-based classifiers (Table 2). The classification framework categorized individuals into three ACE exposure levels: no ACEs, one ACE, and two or more ACEs. The models assessed included traditional multiclass classifiers Logistic Regression, Linear Discriminant Analysis, Decision Tree, Random Forest, Support Vector Machine, Gradient Boosting, K-Nearest Neighbors as well as state-of-the-art ensemble methods such as Extreme Gradient Boosting (XGBoost), Light Gradient Boosting Machine (LightGBM), and CatBoost.Random Forest achieved the highest accuracy of 82% compared to all the models and demonstrated stronger performance across the precision and accuracy classes, identifying individuals with 2 or more ACEs. We conducted a five-fold cross-validation that demonstrated consistent performance across folds (mean accuracy = 0.821, standard deviation = 0.002), indicating model stability and robustness. Decision Tree, XGBoost, and Support Vector Machine each achieved 74% accuracy, while Logistic Regression reached 72% accuracy. Other ensemble methods, including Light Gradient Boosting Machine and CatBoost, achieved accuracies ranging from 70% to 73%. The superior performance of Random Forest suggests that ACE prediction involves complex, non-linear interactions that ensemble tree-based methods can better capture than traditional linear approaches.

## 4. Discussion

This study demonstrates the feasibility of using machine learning approaches to predict ACE exposure from observable family environment characteristics, achieving 82% accuracy with Random Forest modeling. The identification of specific, modifiable family characteristics as strong predictors provides concrete targets for prevention efforts and early intervention programs, representing a significant advancement toward proactive rather than reactive approaches to childhood trauma prevention.

The emergence of child sex (female) as the strongest predictor, despite similar overall ACE prevalence between genders, reveals complex underlying patterns that warrant careful interpretation. This finding may reflect gender-specific vulnerability patterns, with girls showing elevated risk for certain types of ACEs, particularly interpersonal trauma, and different manifestation patterns following ACE exposure (Hughes et al., 2017). Additionally, girls may have different reporting patterns or experience differential detection by caregivers and professionals (Hillis et al., 2004).

These findings should be interpreted within the context of family systems theory, recognizing that many family environment indicators in our model (such as food insufficiency, chronic school absenteeism) represent interconnected aspects of family adversity rather than independent causal factors. This approach quantifies clustering patterns of adversity within family systems, consistent with decades of ACE research documenting the co-occurrence of adverse experiences and the importance of socioeconomic factors in shaping child development outcomes.

The superior performance of ensemble methods supports this interpretation, suggesting that family environments involve complex, non-linear interactions that require sophisticated analytical approaches to fully capture. Rather than establishing direct causation, this work provides empirical foundation for early warning systems that can identify children embedded in concerning patterns of family adversity, offering opportunities for preventive intervention before ACE exposure accumulates.

Food insufficiency emerged as another major predictor, aligning with existing literature identifying food insecurity as a marker of broader family instability, including financial stress, parental mental health concerns, and increased risk of family conflict (Gundersen & Ziliak, 2015; Chilton et al., 2017).

The poor predictive performance for children with single ACEs (F1 = 0.55) represents both a significant limitation and a potentially meaningful finding. This pattern may suggest that children with one ACE represent a heterogeneous group with characteristics that overlap both no-ACE and multiple-ACE categories, making accurate classification particularly difficult. However, this hypothesis requires further investigation to determine whether this reflects genuine population heterogeneity or limitations in the feature set and modeling approach used. The model’s excellent performance in identifying children with no ACEs but moderate performance for multiple ACEs suggests it would be most useful for confirming low risk rather than identifying high-risk cases where intervention would be most beneficial.

Several implementation challenges must be addressed before clinical deployment. The systematic selection bias resulting from 52.2% data loss, with bias toward stable families (31% vs. 38% ACE prevalence in analytical vs. full sample), significantly limits generalizability to the most vulnerable populations, precisely those who would most benefit from early identification and intervention. This bias may result in models that perform well in research settings but poorly in real-world clinical environments where high-risk families are more commonly encountered. Furthermore, the model requires comprehensive family environment assessment that may not be feasible in all clinical settings due to time, resource, or training limitations.

The confusion matrix analysis reveals specific clinical utility concerns beyond overall accuracy metrics. While the model’s 98.1% sensitivity for identifying children with no ACEs provides reassurance about low-risk classification, the high false negative rates for children with ACEs present serious clinical challenges. Specifically, 55.4% of children with single ACE exposure and 43.5% of children with multiple ACEs are misclassified as having no ACE exposure. In clinical settings, these false negatives represent missed opportunities for early intervention when children could most benefit from support services.

The model’s excellent performance in identifying children with no ACEs (98.1% sensitivity) occurs precisely where clinical intervention is least needed, while moderate performance for high-risk cases (53.2% sensitivity for multiple ACEs) occurs where accurate identification is most crucial. This performance pattern suggests the model would be most useful for confirming low-risk status in population screening rather than identifying high-risk cases requiring intervention.

These findings align with decades of ACE research documenting the clustering of adverse experiences within families and the importance of socioeconomic factors and family functioning (Scully et al., 2019). However, this study advances the field by quantifying the relative importance of different characteristics and demonstrating collective predictive power through machine learning approaches. The superior performance of ensemble methods suggests that family systems involve complex interactions requiring sophisticated analytical approaches to fully understand, consistent with systems theory perspectives on family functioning (Walsh, 2016).

Several critical research priorities emerge from this analysis. Longitudinal validation studies are essential to address the limitations of cross-sectional data, as prospective designs would enable validation of predictive models over time and provide understanding of developmental trajectories leading to adverse experiences (Evans et al., 2013; Shonkoff et al., 2012).

Future research must specifically focus on vulnerable populations that were underrepresented in this analysis due to systematic survey non-response patterns. Studies targeting high-risk communities, families experiencing homelessness, those involved with child protective services, and other marginalized populations are essential to understand model performance where it would be most clinically valuable. Integration of qualitative methods through mixed-methods approaches can provide essential context for understanding prediction failures and improving model development. Incorporating lived experiences of families navigating economic hardship, health challenges, and adversity would provide crucial insights into mechanisms through which ACEs operate in diverse family contexts and guide creation of more culturally relevant and effective interventions (Burke Harris, 2018). Finally, rigorous implementation research examining real-world deployment of predictive models is essential before widespread clinical adoption, including comprehensive cost-effectiveness analyses, evaluation of unintended consequences, assessment of provider and family acceptance, and systematic examination of how predictive information influences clinical decision-making and family outcomes.

The implications for vulnerable populations extend beyond simple accuracy concerns. Features that predict ACE exposure in stable families may not transfer to more vulnerable populations. For example, regular school attendance patterns or consistent communication styles may be less reliable indicators in families facing homelessness, recent immigration, or severe poverty. The excluded populations likely include families experiencing housing instability, frequent relocation, limited English proficiency, severe mental health challenges, or involvement with child protective services—precisely those at highest risk for ACE exposure who would most benefit from early identification.

## 5. Limitations of the Study

This study has several important limitations that must be considered when interpreting results and planning implementation. The cross-sectional design prevents establishment of causal relationships between family environment factors and ACE exposure, allowing only for identification of associations and predictive relationships at a single time point.

Systematic selection bias represents a critical limitation, as the 52.2% data loss systematically excludes the most vulnerable populations who would most benefit from early identification. The excluded populations likely include families experiencing housing instability, frequent relocation, limited English proficiency, severe mental health challenges, or involvement with child protective services. This creates a fundamental mismatch between our training population (stable families participating in national surveys) and target clinical populations (high-risk families with chaotic circumstances or institutional distrust). Consequently, our 82% research accuracy may substantially overestimate real-world clinical performance.

Performance limitations, particularly the substantially lower predictive performance for the single ACE category (F1 = 0.55) compared to no ACEs (F1 = 0.89) and multiple ACEs (F1 = 0.64), constrain clinical utility for comprehensive risk assessment. The model’s strongest performance in identifying low-risk children occurs precisely where clinical intervention is least needed, while moderate performance for high-risk cases occurs where accurate identification is most crucial.

Methodological circularity exists in using family environment indicators that may themselves represent forms of adversity to predict ACE exposure, limiting causal interpretation while maintaining utility for pattern recognition and early warning system development.

Clinical implementation feasibility remains unproven, as the model requires comprehensive family environment assessment that may not be practical in clinical settings serving vulnerable populations due to resource constraints, cultural barriers, and workflow limitations.

## 6. Conclusions

This study reveals consistent associations between adverse childhood experiences and multiple dimensions of family environment, particularly food security, school absenteeism, and child demographics. Machine learning approaches can predict ACE exposure from observable family characteristics with 82% accuracy, but implementation must carefully address systematic selection bias, ethical considerations, and performance limitations.

The identification of child sex (female) as the primary predictor, despite similar overall ACE prevalence, suggests complex gender-specific patterns requiring further investigation. The notably lower predictive performance for children with single ACEs (F1 = 0.55) compared to no ACEs (F1 = 0.89) and multiple ACEs (F1 = 0.64) indicates important limitations for clinical screening applications.

Key findings emphasize that ACEs are embedded in families’ daily conditions, with food insecurity and irregular school attendance serving as both symptoms and contributors to adversity (Gundersen & Ziliak, 2015; Bethell et al., 2014). The cumulative nature of these stressors highlights the need for systemic interventions addressing underlying conditions shaping children’s developmental environments.

While these results represent an important step toward evidence-based ACE prevention, rigorous implementation research and longitudinal validation are essential before clinical deployment (Evans et al., 2013; Shonkoff et al., 2012). Success will require careful attention to ethical considerations, equity concerns, systematic biases inherent in survey-based models, and comprehensive evaluation of clinical utility in real-world settings where vulnerable families are commonly encountered.

## Figures and Tables

**Table 1:** Summary statistics of ACEs among U.S. children, NSCH 2018–2020.

ACE Exposure	Est. % U.S. Children	Interpretation
0	69	Most children in the study did not experience ACEs.
1	19	Approx. 1 in 5 (20%) experienced 1 ACE.
2	12	Fewer children experienced 2 ACEs.

**Table 2:** Comparative analysis of various machine learning models’ predictive accuracy for family environments based on ACEs.

Model Type	Precision	Recall	F1 Score	Accuracy
Multiclass Classifiers				
Logistic regression				
No_ACEs	0.74	0.97	0.84	
1 ACE	0.42	0.05	0.09	
2or more ACE	0.52	0.34	0.41	
				0.72
Linear Discriminant Analysis				
No_ACEs	0.75	0.96	0.84	
1_ACEs	0.33	0.05	0.08	
2ormore_ACEs	0.49	0.37	0.42	
				0.71
Decision Tree				
No_ACEs	0.85	0.82	0.83	
1_ACE	0.49	0.53	0.51	
2ormore_ACEs	0.53	0.55	0.54	
				0.74
Random Forest				
No_ACEs	0.81	0.98	0.89	
1_ACE	0.88	0.40	0.55	
2ormore_ACEs	0.81	0.53	0.64	
				0.82
K-Nearest Neighbors				
No_ACEs	0.72	0.99	0.83	
1_ACE	0.35	0.05	0.09	
2ormore_ACEs	0.58	0.12	0.20	
				0.70
Gradient Boosting Machine				
No_ACEs	0.74	0.97	0.84	
1_ACE	0.40	0.05	0.09	
2ormore_ACEs	0.54	0.31	0.39	
				0.72
Support Vector Machine				
No_ACEs	0.75	0.98	0.85	
1_ACE	0.67	0.11	0.19	
2ormore_ACEs	0.68	0.35	0.46	
				0.74
XGBoost				
No_ACEs	0.76	0.96	0.85	
1_ACE	0.54	0.18	0.27	
2ormore_ACEs	0.65	0.39	0.48	
				0.74
Advanced Tree Based Classifiers				
LightGBM				
No_ACEs	0.75	0.97	0.84	
1_ACE	0.53	0.09	0.16	
2ormore_ACEs	0.58	0.35	0.44	
				0.73
CatBoost				
No_ACEs	0.74	0.97	0.84	
1_ACE	0.44	0.05	0.09	
2ormore_ACEs	0.56	0.32	0.41	
				0.72

## Data Availability

Datasets are available upon request from the CAHMI Data Resource Center for Child and Adolescent Health at www.childhealthdata.org and can also be obtained by contacting the corresponding author.

## Abbreviations

The following abbreviations are used in this manuscript:

ACE	Adverse Childhood Experiences
NSCH	National Survey of Children’s Health
AUC	Area under the curve
HRSA	Health Resources and Services Administration
MCHB	Maternal and Child Health Bureau
PTSD	Post Traumatic Stress Disorders
ROCAUC	Receiver Operating Characteristic—Area Under the Curve
